# New Directions in Dietary Restriction: Remembering Edward Masoro

**DOI:** 10.1093/geroni/igab046.439

**Published:** 2021-12-17

**Authors:** Arlan Richardson

## Abstract

In 1935, Clive McCay reported that severe restriction of food increased the lifespan of male rats. In the following four decades, several laboratories replicated this observation with less sever restrictions, which will be referred to as dietary restriction (DR). However, there were concerns even in the aging community in the 1970s as to whether DR increased lifespan by retarding aging. It was the research of two former Kleemeier Awardees, Edward Masoro and Roy Walford, that conclusively demonstrated in the 1980s that DR retarded aging resulting in improved healthspan and reduced pathology. Ed Masoro’s research was focused on lipid metabolism when he was invited to attend a workshop on metabolism and aging in 1969. His interest in aging was piqued such that the more he learned about aging, the more interested he became. In a subsequent workshop in 1973, Ed heard Morris Ross describe his research on restricting food intake on cancer and longevity. Ed was impressed that a relatively simple manipulation had such dramatic effects, and he decided to focus his research on DR. After an extensive review of the DR literature up to the 1970s, Ed established the 40% restriction paradigm, which is used in almost all DR studies to date. Ed’s group was the first to study aging and DR under barrier conditions which he established at San Antonio. Over the next two decades, Ed would direct a Program Project that showed DR had a dramatic effect on most age-related pathologies and improved many physiological functions. Studying the restriction of fat, protein, micronutrients, Ed came to the conclusion that total calories consumed was a key factor in the effect of DR on longevity. His group was the first to show that DR significantly reduced circulating levels of glucose and insulin, which was subsequently shown to occur because of increased insulin sensitivity and is now recognized as a hallmark of DR and potentially important in the anti-aging action of DR. Ed was chair of the Biological Sciences Section of GSA in 1979 and President in 1995. This session is dedicated to Edward Masoro who passed away on July 11, 2020 at the age or 95. Dr. Masoro was president in 1995 and BS chair in 1979, Clive McCay was President in 1949.

